# Visualization of Sterol-Rich Membrane Domains with Fluorescently-Labeled Theonellamides

**DOI:** 10.1371/journal.pone.0083716

**Published:** 2013-12-27

**Authors:** Shinichi Nishimura, Kumiko Ishii, Kunihiko Iwamoto, Yuko Arita, Shigeki Matsunaga, Yoshiko Ohno-Iwashita, Satoshi B. Sato, Hideaki Kakeya, Toshihide Kobayashi, Minoru Yoshida

**Affiliations:** 1 Chemical Genomics Research Group, RIKEN Center for Sustainable Resource Science, Saitama, Japan; 2 Division of Bioinformatics and Chemical Genomics, Graduate School of Pharmaceutical Sciences, Kyoto University, Kyoto, Japan; 3 Lipid Biology Laboratory, RIKEN, Saitama, Japan; 4 Laboratory of Aquatic Natural Products Chemistry, Graduate School of Agricultural and Life Sciences, The University of Tokyo, Tokyo, Japan; 5 Faculty of Pharmacy, Iwaki Meisei University, Iwaki, Fukushima, Japan; 6 Research Center for Low Temperature and Materials Sciences, Kyoto University, Kyoto, Japan; Johns Hopkins School of Medicine, United States of America

## Abstract

Cholesterol plays important roles in biological membranes. The cellular location where cholesterol molecules work is prerequisite information for understanding their dynamic action. Bioimaging probes for cholesterol molecules would be the most powerful means for unraveling the complex nature of lipid membranes. However, only a limited number of chemical or protein probes have been developed so far for cytological analysis. Here we show that fluorescently-labeled derivatives of theonellamides act as new sterol probes in mammalian cultured cells. The fluorescent probes recognized cholesterol molecules and bound to liposomes in a cholesterol-concentration dependent manner. The probes showed patchy distribution in the plasma membrane, while they stained specific organelle in the cytoplasm. These data suggest that fTNMs will be valuable sterol probes for studies on the role of sterols in the biological membrane under a variety of experimental conditions.

## Introduction

The spatiotemporal regulation of cellular molecules is fundamental to orchestrate the robust cellular systems. Genetically-engineered probes for the analysis of protein behavior, e.g., green fluorescent protein (GFP)-fusion proteins, have created a powerful means of visualizing the location and dynamic behaviors of proteins in cells. In contrast, lipid molecules are not directly encoded by the genome, hence genetic methods cannot be applied for observing their subcellular localization. To detect lipid molecules by fluorescent microscopy, fluorescent lipid analogs and lipid-binding molecules have been used. In the case of sterol molecules, intrinsically fluorescent sterols and fluorescently-labeled cholesterol derivatives are useful mimics for detecting the localization of sterol molecules in living cells[1]. Filipin[2], [3], a polyene antibiotic, is a traditional sterol marker, while sterol-interacting proteins such as perfringolysin O (PFO, also known as *θ*-toxin), a bacterial cytolysin[4], have been successfully used to detect sterol molecules in fixed cells. However, it should be noted that chemically-modified sterol analogs do not retain all of the properties of the parental molecules. In addition, the molecular size of sterol-binding proteins is much larger than the size of sterol molecules and may induce alterations in membrane organization. Even filipin, the most commonly used sterol marker, has a critical limitation because of its rapid photobleaching, and it is also known to induce cellular damage[1]. These properties have prevented its wide application to live cell imaging with a few exceptions[5], [6]. For analyzing the location and function of membrane lipids, easy-to-use chemical probes, particularly for live cell imaging, remain in high demand. Here, we report theonellamides (TNMs) as alternative sterol probes. Fluorescently-labeled TNMs (fTNMs) are effective probes for the visualization of intracellular and plasma membrane sterol molecules in fixed cells, and for plasma membrane sterol molecules in living cells.

## Results and Discussion

**Figure 1:**
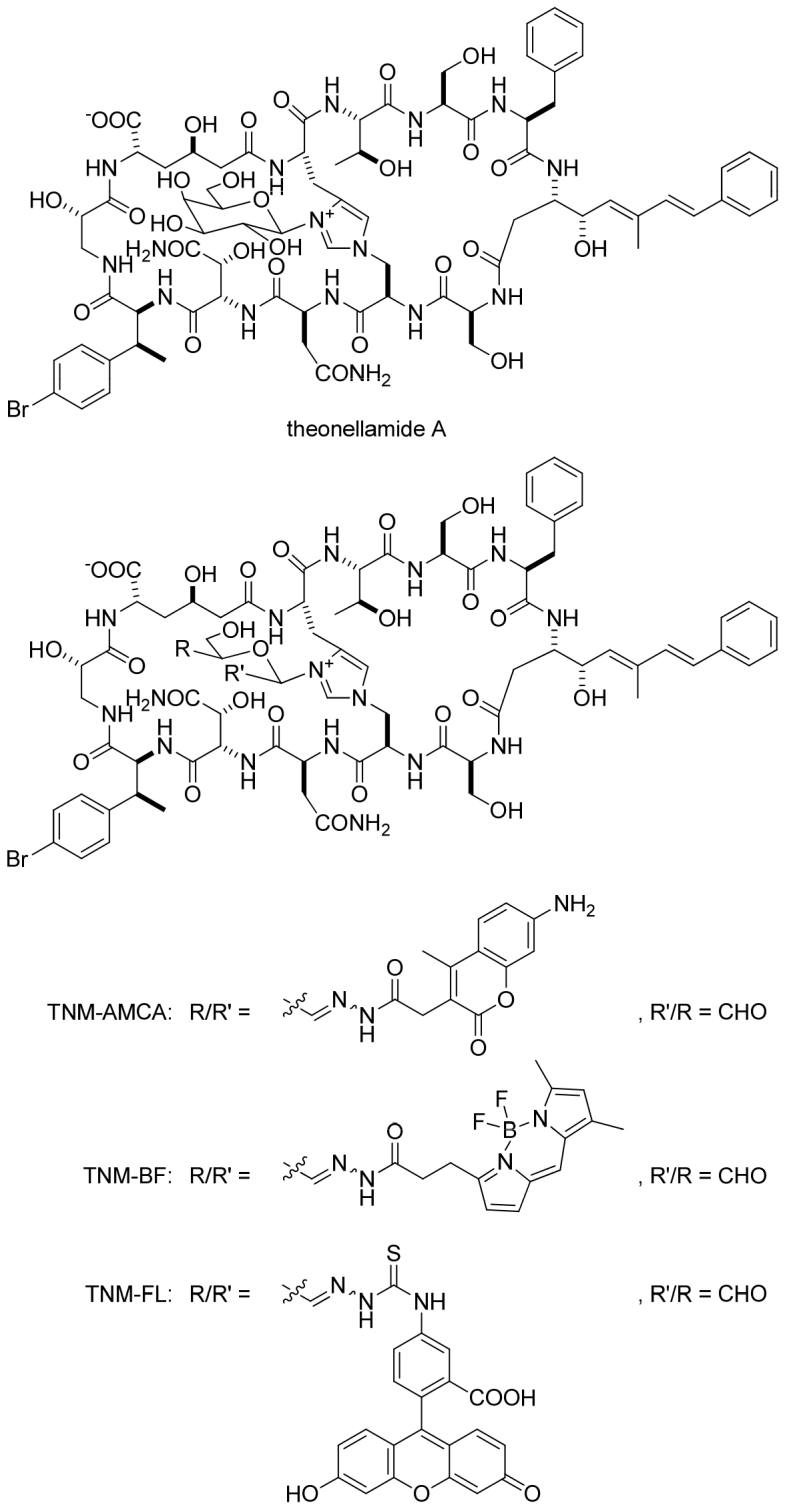
Chemical structures of theonellamide A and fluorescently-labeled derivatives.

**Figure 2:**
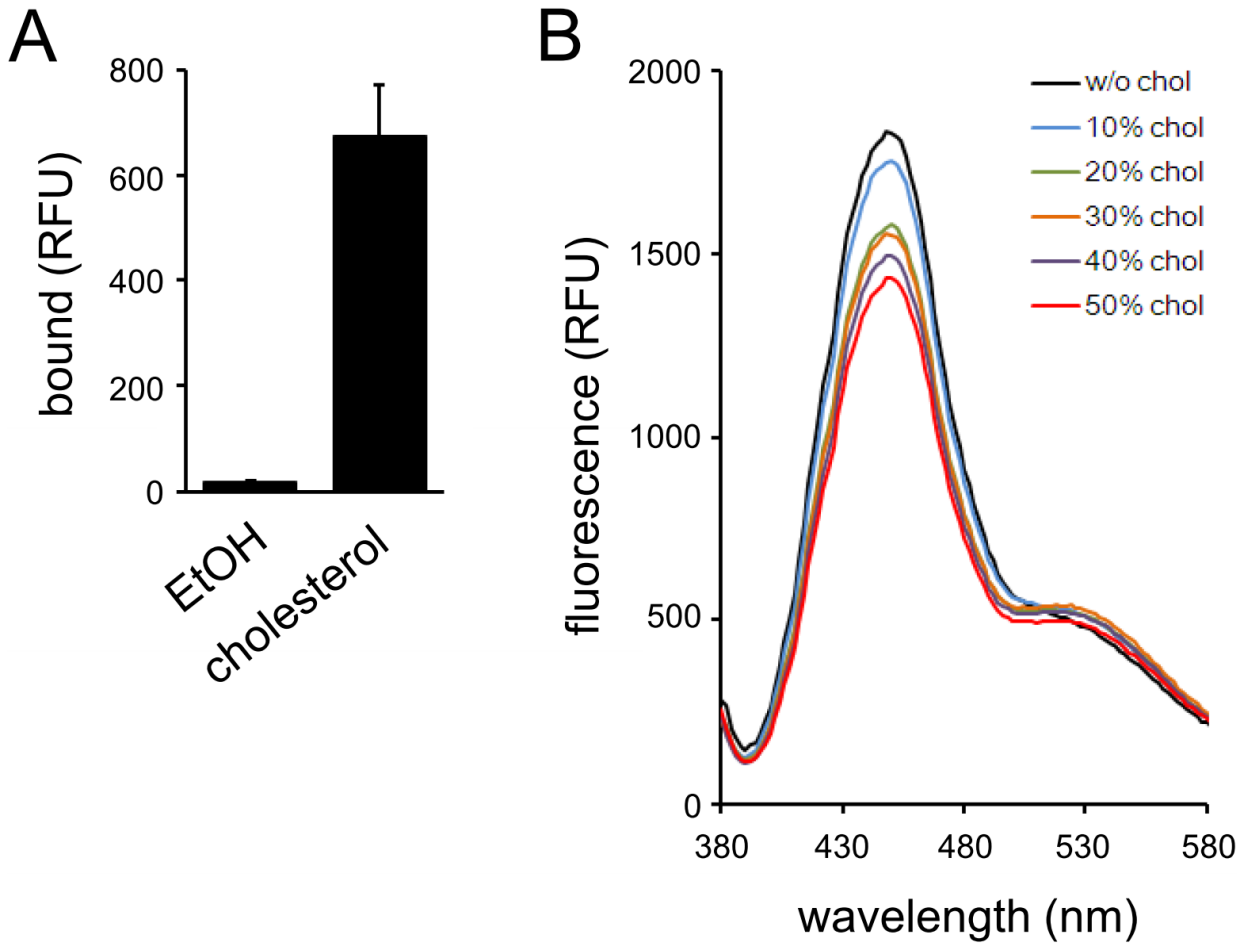
Binding of TNM-AMCA to cholesterol. (A) Binding of TNM-AMCA to cholesterol molecules was examined on a hydrophobic plate. (B) TNM-AMCA was mixed with liposomes containing various concentrations of cholesterol and NBD-PE, and fluorescent spectra (ex = 345 nm) were recorded. Data represent means of three (A) or four (B) independent experiments. Error bars, s.d.

TNMs are bicyclic peptides discovered from marine sponges of the genus *Theonella* (**Figure 1**) [7]–[10]. They exhibit potent antifungal activity and inhibit yeast cell growth as a consequence of binding to ergosterol [11], [12]. Fluorescently-labeled TNMs (fTNMs) possessing aminomethyl coumarin acetic acid (AMCA) or BODIPY-FL functionality (TNM-AMCA and TNM-BF respectively, **Figure 1**) recognized membrane ergosterol both in fission and budding yeast cells, and the obtained fluorescence images were similar to those of filipin. We reported that TNM-BF recognizes 3β-hydroxysterols; the affinity was not affected by the degree of unsaturation in the ring B of sterols [12]. In addition, recently, a non-labeled natural product TNM-A was shown to bind to liposomes containing 10 mol% ergosterol or cholesterol [13]. These results prompted us to test whether fTNMs could recognize membrane sterols in mammalian cells. First we examined the affinity of TNM-AMCA to cholesterol, the most major sterol molecule in mammalian cells (**Figure 2A**). The binding was examined on a hydrophobic plate, where cholesterol solution was dropped and the solvent was evaporated. TNM-AMCA bound to plates where cholesterol is fixed, whereas binding to the control plate was hardly observed. This indicated that TNM-AMCA directly recognizes cholesterol.

Next, to investigate the binding of TNM-AMCA to cholesterol-containing membranes, the fluorescence of TNM-AMCA in the presence of liposomes consisting of 1-palmitoyl-2-oleoyl-*sn*-glycero-3-phosphocholine (POPC) and a variety of concentration of cholesterol was measured. The liposomes contained 1 mol% of *N*-(7-nitrobenz-2-oxa-1,3-diazol-4-yl)-1,2-dioleoyl-*sn*-glycero-3-ethanolamine (NBD-PE); if TNM-AMCA binds to liposomes and TNM-AMCA and NBD-PE are closely located, the fluorescence of TNM-AMCA would decrease since the fluorescence spectrum of AMCA and the absorbance spectrum of NBD is overlapped. As expected, the fluorescence of TNM-AMCA decreased in a cholesterol concentration-dependent manner, although increase of NBD fluorescence was hardly observed (**Figure 2B**). To investigate the contribution of NBD-PE to the decrease of TNM-AMCA fluorescence, we measured the fluorescence of TNM-AMCA when incubated with liposomes without NBD-PE (**Figure S1**). Even in the absence of NBD-PE, the fluorescence of TNM-AMCA slightly decreased when more than 20 mol% cholesterol was included in the liposomes. These results indicate that the decrease of TNM-AMCA fluorescence observed in **Figure 2B** was due to both the energy transfer from TNM-AMCA to NBD-PE, which might be too small to exhibit enough emission of NBD, and the quenching of TNM-AMCA fluorescence with some unknown mechanism, e.g. aggregation. These results implied that TNM-AMCA would bind to membrane domains with higher sterol concentration.

**Figure 3:**
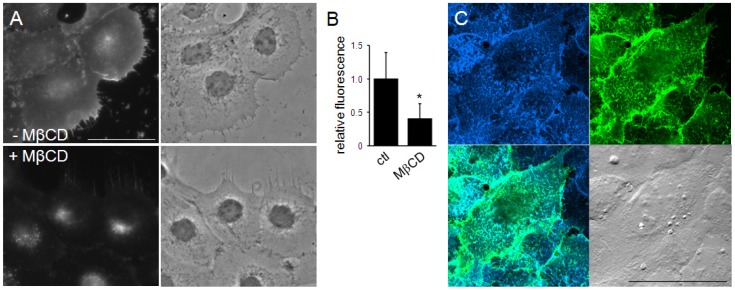
Cell surface labeling with TNM-AMCA. (A, B) A431 cells were fixed and labeled with TNM-AMCA. The effect of sterol extraction with MβCD was examined. Fluorescent images (A) and the quantification of the fluorescence (B) are shown. Relative fluorescence corresponds to the mean ± SD for more than 35 cells in two independent experiments. Asterisk indicates statistically significant difference (p<1×10^−10^). (C) HeLa cells were co-stained with TNM-AMCA (blue) and BC*θ* (green). Merged fluorescence (lower left) and DIC (lower right) images are also shown. Cells were fixed, incubated with BC*θ* followed by streptavidin-Alexa Fluor 488 treatment, and then labeled with TNM-AMCA. Scale bars, 50 µm.

Cholesterol is one of the most abundant lipid species in the plasma membrane of mammalian cells. Next, we investigated the possibility that TNM-AMCA could probe membrane sterols including cholesterol in a cell. As expected, TNM-AMCA stained the cell surface of cultured human cells (**Figure 3A**). Removal of the cell surface sterols by pre-treating cells with methyl-*β*-cyclodextrin [14] resulted in a significantly decreased fluorescent signal (**Figure 3A, B**), suggesting that TNM-AMCA recognizes plasma membrane sterols. We compared the fluorescence pattern of TNM-AMCA and BC*θ* (biotinylated derivative of the Carlsberg protease-nicked PFO/*θ*-toxin), one of the PFO/*θ*-toxin derivatives [4], [15]. PFO/*θ*-toxin is a protein toxin produced by *Clostridium perfringens* that recognizes cholesterol-rich membrane domains; BC*θ* is a non-toxic derivative of PFO/*θ*-toxin that retains the ability to bind to cholesterol-rich membrane microdomains [16]. Under confocal laser microscopy, dotted fluorescent signals of TNM-AMCA were observed. The staining pattern of TNM-AMCA on the cell surface was almost the same as that of BC*θ* (**Figure 3C**). These results demonstrated that staining with fTNM is a convenient means of detecting cell surface sterol molecules in fixed cells.

**Figure 4:**
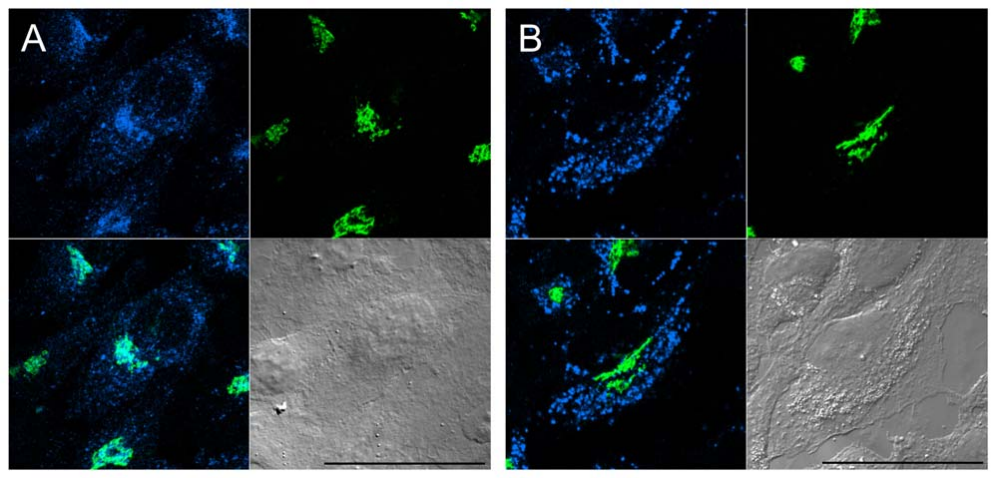
Detection of intracellular sterols with TNM-AMCA. HeLa cells were cultured in the absence (A) or presence (B) of U18666A, fixed, permeabilized and labeled with anti-GM130 antibody (green) and TNM-AMCA (blue). Merged fluorescence (lower left) and DIC (lower right) images are also shown. Scale bars, 50 µm.

Inside mammalian cells, a graded level of free cholesterol is distributed in various membrane organelles, with the higher accumulation in domains of post-Golgi membranes [17]. To explore the possible use of TNM-AMCA as a probe for detecting intracellular sterols, we examined the localization of TNM-AMCA in fixed and digitonin-permeabilized cells. TNM-AMCA exhibited dotted fluorescent signals, which partially overlapped with the *cis*-Golgi marker, GM130 [18] (**Figure 4A**). 3-β-[2-(diethylamino)ethoxy]androst-5-en-17-one (U18666A) is known to accumulate cholesterol in the late endosome, which is a similar phenotype to that observed in Niemann-Pick type C cells [19],[20]. U18666A treatment changed the staining pattern of TNM-AMCA; TNM-AMCA again stained dotted structures, however, the GM130 signal did not merge with the TNM-AMCA signal (**Figure 4B**). Late endosome is rich in lysobisphosphatidic acid (LBPA). When cells were treated with U18666A, LBPA visualized by its antibody and TNM-AMCA partially overlapped (**Figure S2**): some vesicular structures were recognized by both the LBPA antibody and TNM-AMCA, whereas some fluorescent signals of the LBPA antibody and those of TNM-AMCA contacted each other. The fluorescent function, AMCA-hydrazide, itself gave no signal regardless of U18666A treatment (**Figure S2**). Taken together, these results indicate that TNM-AMCA recognizes intracellular cholesterol molecules. Next, we compared the fluorescent signal with that of a fluorescein ester of polyethylene glycol-derivatized cholesterol (fPEG-Chol) (**Figure S3**) [21]. We reported that fPEG-Chol stained the Golgi apparatus and intracellular small vesicles, while the staining pattern was very similar to that of filipin [22]. The fluorescent region of TNM-AMCA was also labeled by fPEG-Chol. However, fPEG-Chol stained a slightly broader region. This was more apparent in cholesterol-starved cells. When cells were cultured in lipoprotein-deficient medium and treated with lovastatin, the filipin signals [23] and the fPEG-Chol signals (**Figure S3**) were largely unaffected. In contrast, the area stained with TNM-AMCA was apparently smaller, suggesting that TNM-AMCA distinguishes subtle changes in sterol status, e.g., local concentration. Thus, TNM-AMCA may serve as a novel chemical probe with a unique mode of sterol recognition.

**Figure 5:**
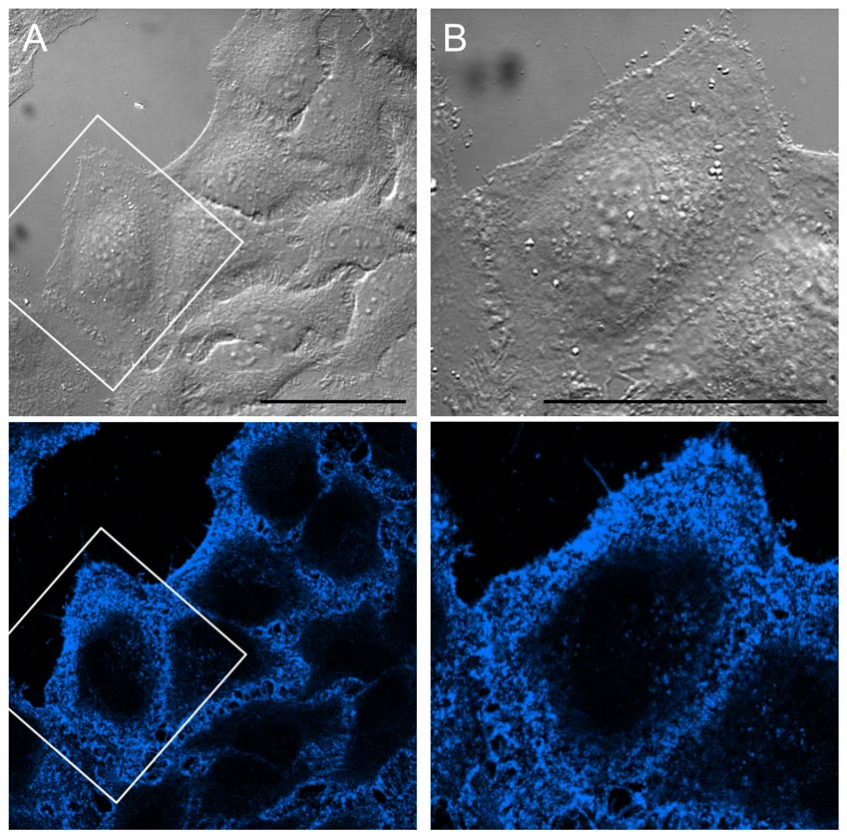
Visualization of live-cell surface sterols with TNM-AMCA. HeLa cells were treated with TNM-AMCA (1 µM) on ice for 30 min. After washing out excess probe molecules, the cells were observed by microscopy. Magnified images are shown in B. Scale bars, 50 µm.

The high selectivity of TNM-AMCA in recognizing sterols in fixed cells prompted us to test whether TNM-AMCA can be applied for labeling membrane sterols in living cells. We treated HeLa cells with TNM-AMCA, following which the free TNM-AMCA was washed out. We successfully detected patchy and punctuate fluorescent signals on the cell surface (**Figure 5** **, Figure S4**). Again, AMCA-hydrazide itself gave negligible fluorescent signals (**Figure S4**). It is likely that TNM-AMCA labeled specific membrane domains with a higher sterol concentration in the plasma membrane. In fact, in fixed cells, TNM-AMCA colocalized well with BC*θ*, which recognizes cholesterol-rich membrane domains (**Figure 3B**) [16]. Detailed analysis of membrane domains recognized by TNMs is under way.

In summary, we have demonstrated that cellular membrane sterols can be visualized with fTNMs. fTNMs bound to liposomes in a manner dependent on the cholesterol concentration, while cellular analysis showed that fTNMs visualized sterol-rich membrane domains in A431 and HeLa cells. fTNMs have several unique properties when compared with other conventional sterol markers. First, fTNMs have versatile fluorescent properties; it is possible to introduce a variety of fluorescent dyes into TNM through its galactose function. We previously reported that fTNM conjugated with BODIPY-FL (TNM-BF) worked well as an ergosterol probe in fission yeast cells [12]. In addition, the fluorescein conjugate, TNM-FL, was also found to recognize cholesterol molecules (**Figure 1** **, Figure S5**). Therefore, it is possible to employ fTNM for co-staining experiments by choosing an appropriate fluorescent group to be conjugated. Second, TNMs can label both cell surface sterols and intracellular sterols, depending on the experimental conditions. We can easily distinguish the cellular location of sterols with fTNMs because of their low membrane permeability. Finally, TNMs have chemical structures distinct from other sterol-binding molecules, implying the presence of unique molecular modes of action. In fact, it is assumed that TNM-A recognizes 3β-hydroxysterols in the shallow area of the membrane [24], which apparently differs from that of polyene antibiotics; for example, amphotericin B forms ion channels in lipid bilayers [25], [26]. A detailed analysis in the binding mode between TNMs and sterols is under way. A high sterol content in eukaryotic cell membranes is thought to influence membrane properties such as permeability, fluidity and microdomain formation [27]. fTNMs with unique properties will enable the fundamental nature of membrane lipids to be revealed.

## Materials and Methods

### Cell and Reagents

A431, A549 and HeLa cells were cultured in Dulbecco's modified Eagle's medium supplemented with 10% fetal calf serum, 100 units/ml penicillin and 100 µg/ml streptomycin. POPC, cholesterol and NBD-PE were purchased from Avanti. M*β*CD was from Wako Pure Chemical Industries (Osaka, Japan). Filipin was from Polysciences (Warrington, PA). Lipoprotein-deficient bovine serum (LPDS) were from Sigma (St. Louis, MO), antibodies were form BD Transduction Laboratories (San Diego, CA). fPEG-Chol and BC*θ* were prepared as described previously [15], [22].

### Preparation of fTNMs

TNM-AMCA was prepared as reported previously [11]. TNM-FL was prepared in a same manner using fluorescein-5-thiosemicarbazide (Molecular Probes). TNM-FL: UV (MeCN:PBS  = 6∶4) *λ*_max_ (*ε*) 279.0 (45,000), 497.0 (61,000) nm; MALDI-TOF-MS (positive) *m/z* 2134.609 [M+H]^+^; HR-ESI-TOF-MS (positive) *m/z* 1067.8259 [M+2H]^2+^ (calcd for C_96/2_H_110/2_^79^Br_1/2_N_19/2_O_31/2_S_1/2_ 1067.8254).

### Binding of fTNMs to lipids

Lipid binding experiment on a hydrophobic plate was examined as described previously [11], [12]. The bound fluorescence of fTNM (ex = 345 nm, em = 450 nm for TNM-AMCA; ex = 490 nm, em = 528 nm for TNM-FL) was measured using a microplate reader SpectraMax M2e (Molecular Devices).

### Binding of fTNMs to liposomes

Small unilamellar vesicles consisting of POPC and cholesterol (0–50 mol%) with or without 1 mol% of NBD-PE were prepared as described previously [28]. TNM-AMCA (1 µM) was mixed with small unilamellar vesicles (100 µM total lipids) in an incubation buffer (20 mM HEPES, pH 7.4, 100 mM NaCl), followed by 30 min incubation. Fluorescence measurements (ex = 345 nm) were performed using a microplate reader SpectraMax M2e (Molecular Devices).

### Microscopy

The specimens were observed using Zeiss LSM 510 confocal microscope equipped with C-Apochromat 63XW Korr objective (Carl Zeiss, Oberkochen, Germany). In Fig. 3A, images were acquired using an Olympus IX81 microscope equipped with LUCPlanFLN 40x objective (Olympus, Tokyo, Japan).

### Cell staining

For staining fixed cells, cells cultured on coverslips were washed in ice-cold PBS, fixed with 3% paraformaldehyde for 10 min at room temperature, and treated with 50 mM NH_4_Cl for 10 min. Cells were then labeled with BC*θ* (10 µg/ml) followed by incubation with Alexa Fluor 488 (or Alexa Fluor 546)-conjugated streptavidin. Then, the specimens were incubated with TNM-AMCA (1 µM) for 1 h, and subjected to microscopy. To label intracelular structures, cells were permeabilized with digitonin (50 µg/ml) in PBS for 5 min after fixation. To detect GM130, the specimens were incubated with anti-GM130 antibody, followed by Alexa Fluor 448 (or Alexa Fluor 546)-conjugated anti-mouse IgG. LBPA was detected using anti-LBPA antibody, followed by Alexa Fluor 546-conjugated anti-mouse IgG. For double staining, cells were reacted with anti-LBPA antibody, followed by incubation with secondary antibody and TNM-AMCA (1 µM). For triple staining, TNM-AMCA (1 µM) or filipin (50 µg/ml) was added when the anti-GM130 antibody was reacted with the specimens and after the secondary antibody was washed out, while fPEG-Chol (1 µM) was added when the secondary antibody was reacted. Staining of live cells with fTNMs were carried out on ice or at 15°C for 15–30 min. After washing out free probes, specimens were observed under microscopy.

### M*β*CD Treatment and cholesterol starvation

Cells on coverslips were washed with medium, followed by treatment with M*β*CD (10 mM) in medium for 15 min at 37°C. After washing out the M*β*CD solution, cells were fixed and stained with TNM-AMCA. The binding of TNM-AMCA was measured using a Metamorph system (Universal Imaging Corp.). The intensity in a circular region with a dimeter of 10 µm per a cell was measured and subjected to statistical analysis. For cholesterol starvation, cells were incubated with lovastatin (4 µM) and mevalonate (100 µM) in DMEM supplemented with 5% LPDS for 1 overnight at 37°C. After washing out the medium, cells were fixed and stained.

## Supporting Information

Figure S1 **Fluorescence of TNM-AMCA in the presence of liposomes.** TNM-AMCA was mixed with liposomes containing various concentrations of cholesterol, and fluorescent spectra (ex = 345 nm) were recorded. Data represent means of four independent experiments.
(TIF)

Figure S2 **Detection of intracellular sterols with TNM-AMCA.** HeLa cells were cultured in the absence (A, C) or presence (B, D, E–F) of U18666A, fixed, permeabilized and labeled with anti-GM130 antibody (A–D, green) or anti-LBPA antibody (E–F, red) and TNM-AMCA (A, B, E, F; blue) or AMCA-hydroazide (C, D; upper left). Merged fluorescent (lower left) and DIC (lower right) images are also shown. A magnified image of E is shown in F. Scale bars, 50 µm.
(TIF)

Figure S3 Triple staining of HeLa cells by TNM-AMCA, fPEG-Chol, and GM130. Cells cultured in conventional DMEM medium (A, B) or cholesterol-starved cells (C, D) were stained. Enlarged images of (A) and (C) are shown in (B) and (D), respectively. White color indicates the co-localization of the three fluorescence. In cholesterol-starved cells, area stained by TNM-AMCA was small. Scale bars, 50 µm.
(TIF)

Figure S4 **Visualization of live-cell surface sterols with TNM-AMCA.** HeLa cells were treated with TNM-AMCA (A, B) or AMCA-hydroazide (C) on ice for 30 min. After excess probe molecules were washed out, cells were observed under microscopy. Magnified images of A are shown in B. Scale bars, 50 µm. (D) A549 cells were treated with TNM-AMCA (blue) and a plasma membrane marker DiIC_16_(3) (red) at 15°C for 15 min. After excess probe molecules were washed out, cells were fixed with PFA and observed under microscopy. Scale bar, 15 µm.
(TIF)

Figure S5 **Labeling of cell surface sterols with TNM-FL.** (A) TNM-FL recognized cholesterol molecules on a hydrophobic plate. Phospholipids tested were not recognized by TNM-FL. Data represent means of three independent experiments. Error bars, s.d. (B–D) HeLa cells were treated with fluorescein-5-thiosemicarbazide (B) or TNM-FL (C, D) on ice for 30 min. After excess probe molecules were washed out, cells were observed under microscopy. Magnified images of C are shown in D. Scale bars, 50 µm.
(TIF)
