# Supplementary figures and images for: Visualization of Sterol-Rich Membrane Domains with Fluorescently-Labeled Theonellamides
Source: PLoS One. 2013 Dec 27;8(12):e83716. doi: 10.1371/journal.pone.0083716 (PMC3873978; doi:10.1371/journal.pone.0083716)

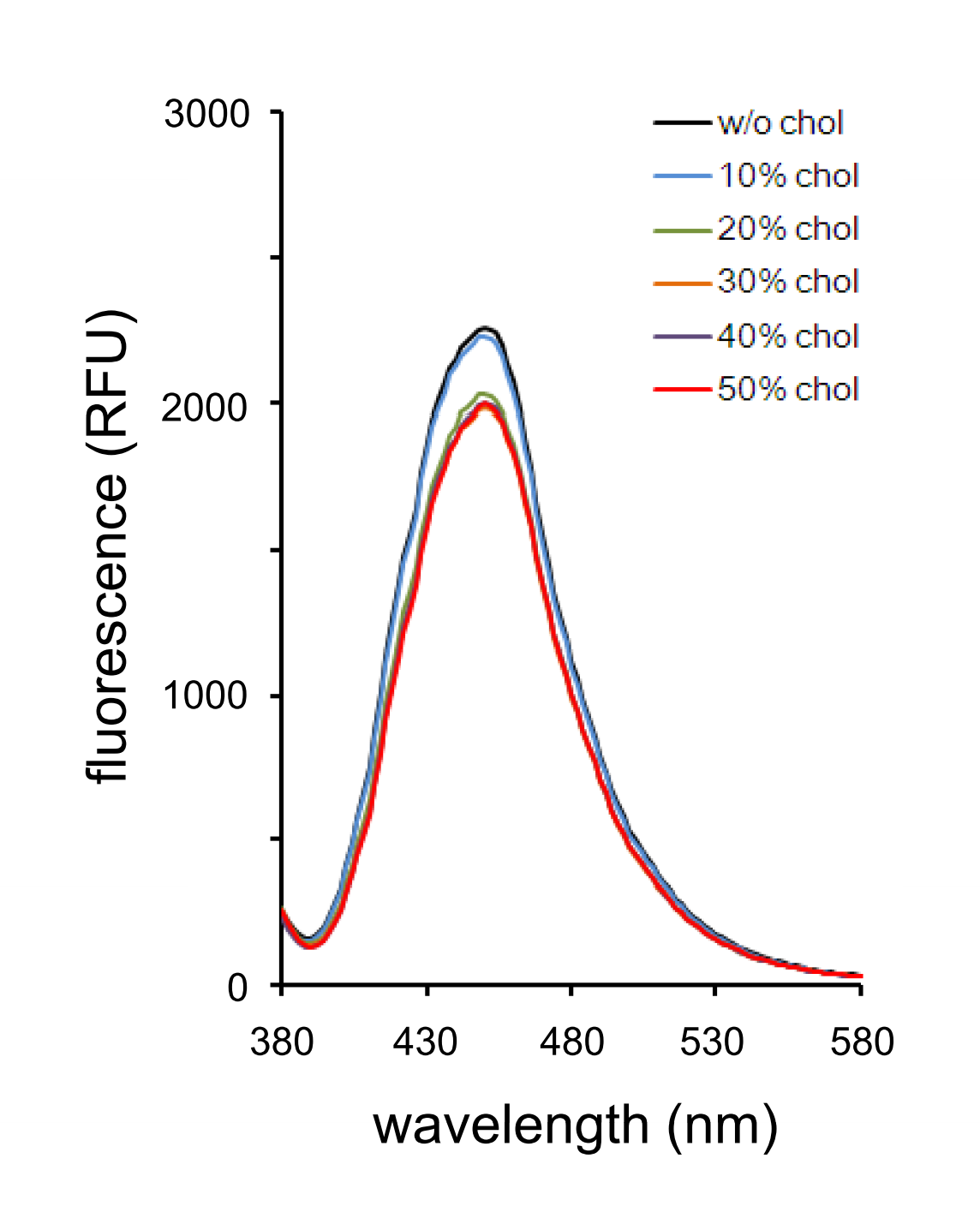

Supplement: Figure S1 — Fluorescence of TNM-AMCA in the presence of liposomes. TNM-AMCA was mixed with liposomes containing various concentrations of cholesterol, and fluorescent spectra (ex = 345 nm) were recorded. Data represent means of four independent experiments. (TIF) [file pone.0083716.s001.tif]

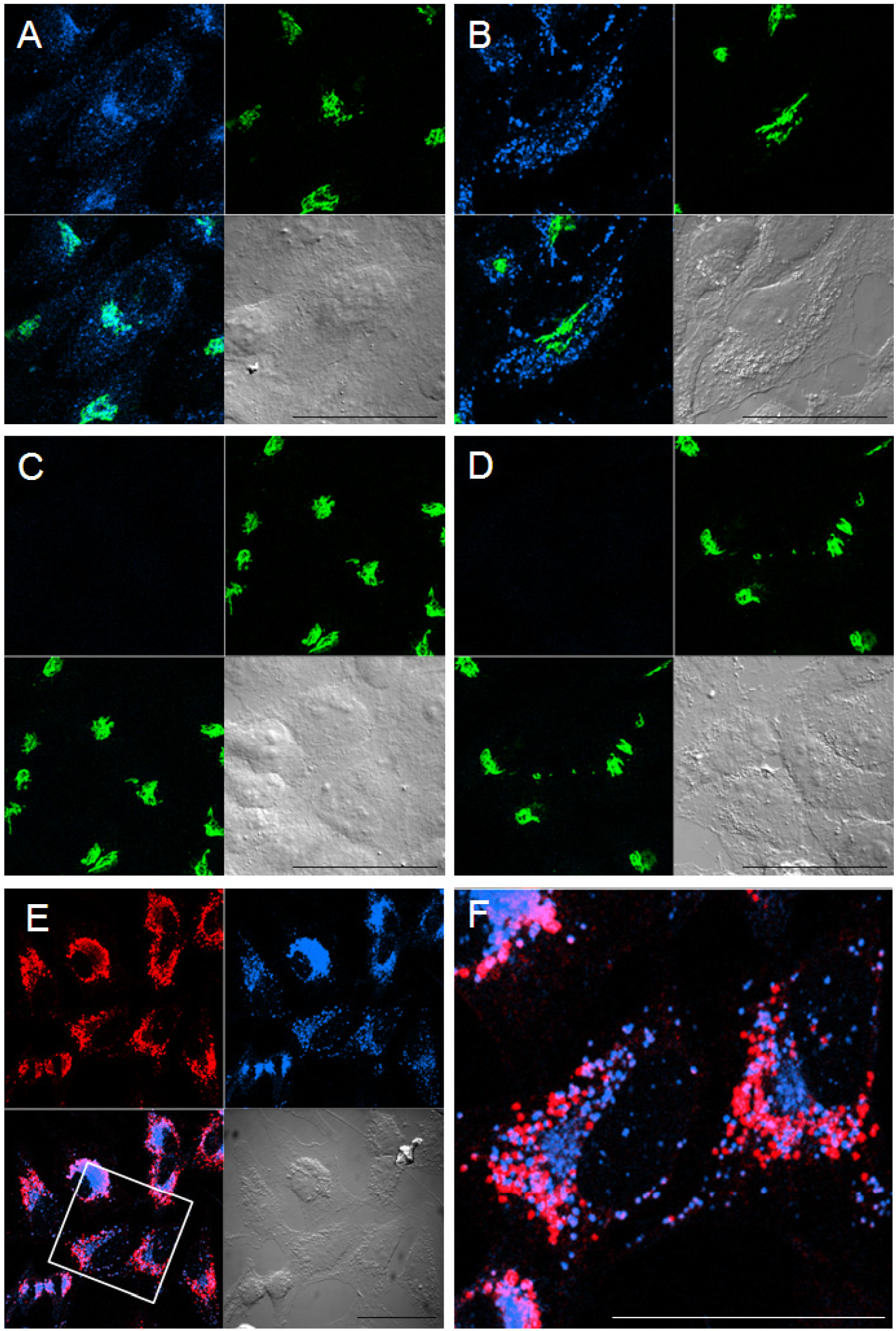

Supplement: Figure S2 — Detection of intracellular sterols with TNM-AMCA. HeLa cells were cultured in the absence (A, C) or presence (B, D, E–F) of U18666A, fixed, permeabilized and labeled with anti-GM130 antibody (A–D, green) or anti-LBPA antibody (E–F, red) and TNM-AMCA (A, B, E, F; blue) or AMCA-hydroazide (C, D; upper left). Merged fluorescent (lower left) and DIC (lower right) images are also shown. A magnified image of E is shown in F. Scale bars, 50 µm. (TIF) [file pone.0083716.s002.tif]

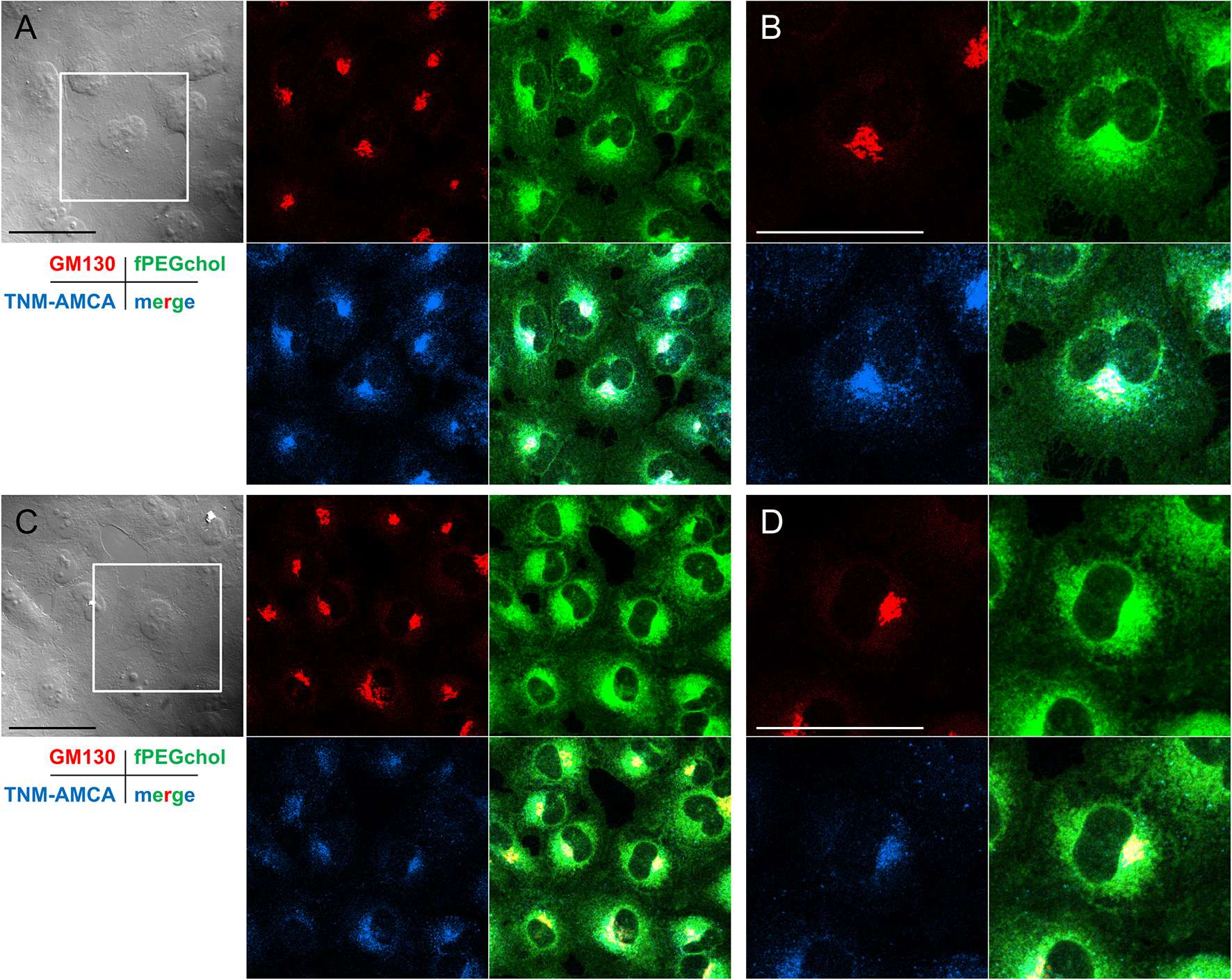

Supplement: Figure S3 — Triple staining of HeLa cells by TNM-AMCA, fPEG-Chol, and GM130. Cells cultured in conventional DMEM medium (A, B) or cholesterol-starved cells (C, D) were stained. Enlarged images of (A) and (C) are shown in (B) and (D), respectively. White color indicates the co-localization of the three fluorescence. In cholesterol-starved cells, area stained by TNM-AMCA was small. Scale bars, 50 µm. (TIF) [file pone.0083716.s003.tif]

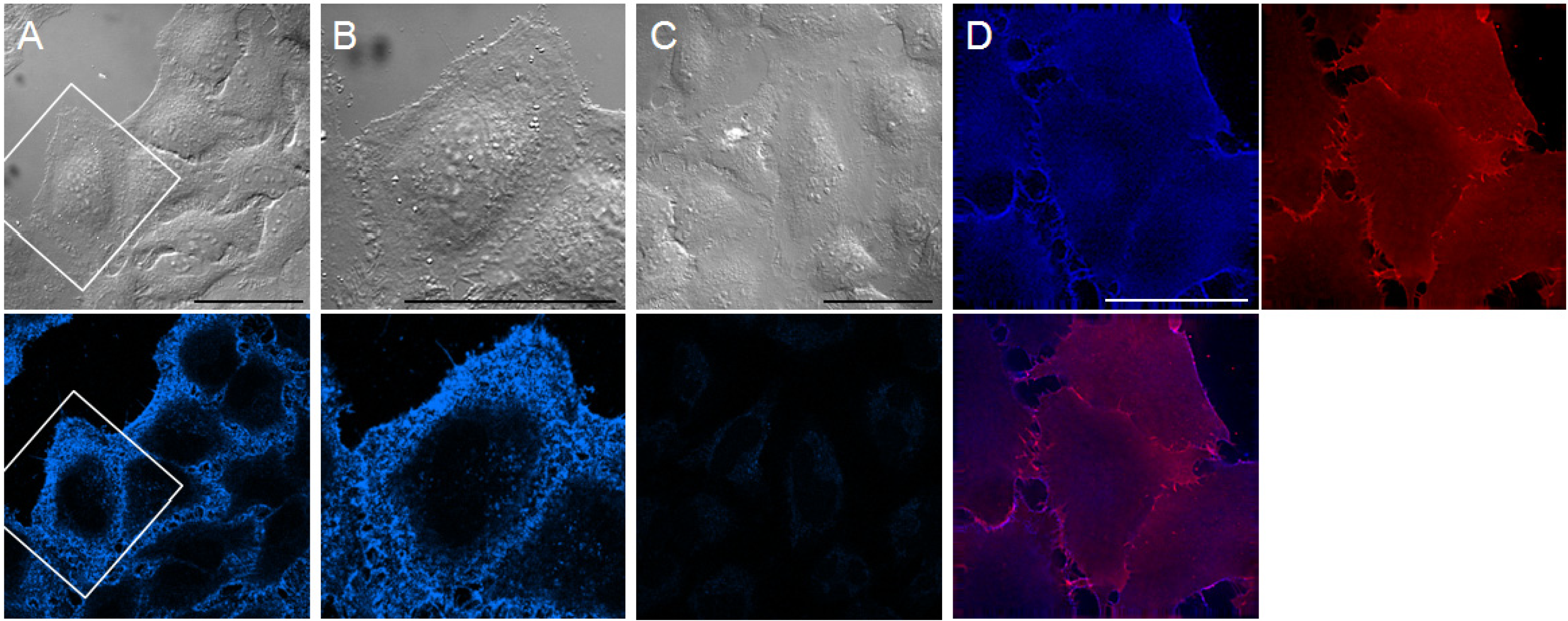

Supplement: Figure S4 — Visualization of live-cell surface sterols with TNM-AMCA. HeLa cells were treated with TNM-AMCA (A, B) or AMCA-hydroazide (C) on ice for 30 min. After excess probe molecules were washed out, cells were observed under microscopy. Magnified images of A are shown in B. Scale bars, 50 µm. (D) A549 cells were treated with TNM-AMCA (blue) and a plasma membrane marker DiIC16(3) (red) at 15°C for 15 min. After excess probe molecules were washed out, cells were fixed with PFA and observed under microscopy. Scale bar, 15 µm. (TIF) [file pone.0083716.s004.tif]

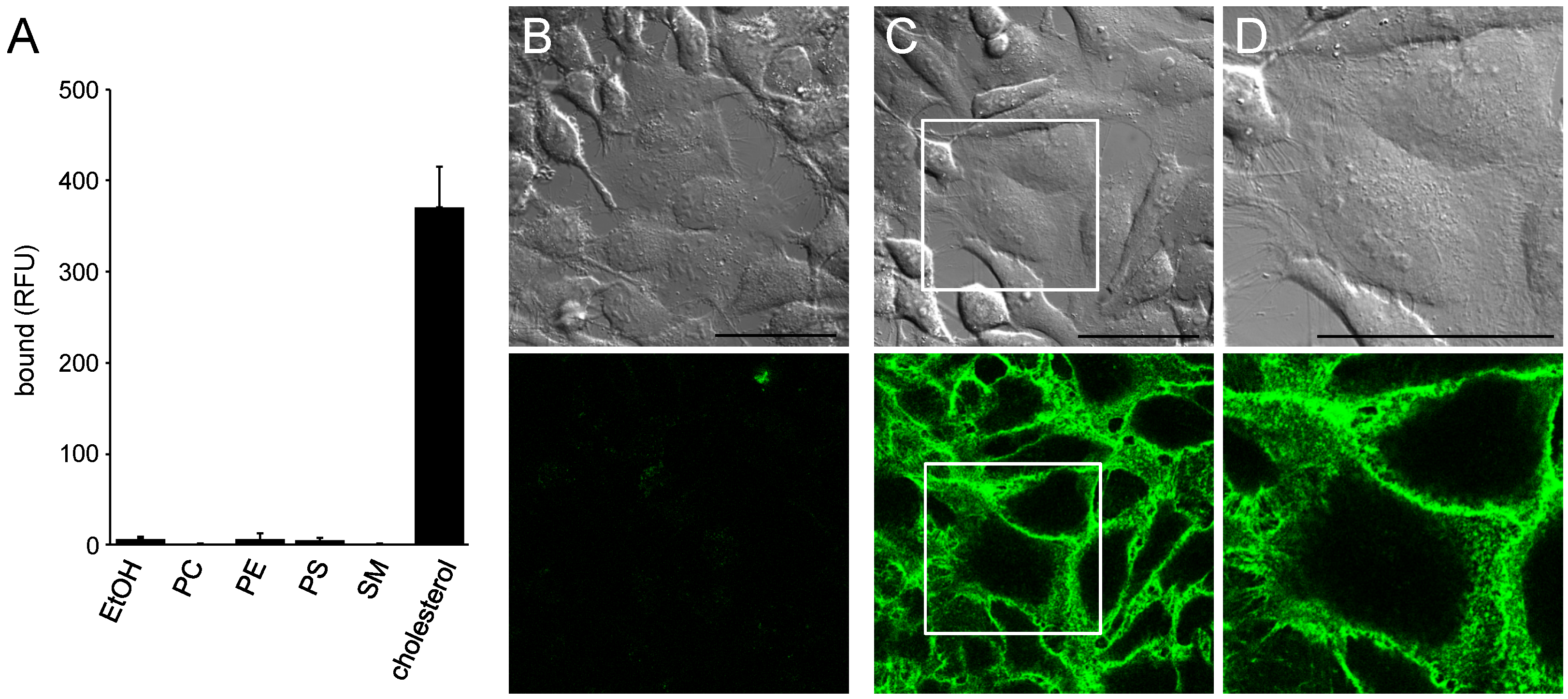

Supplement: Figure S5 — Labeling of cell surface sterols with TNM-FL. (A) TNM-FL recognized cholesterol molecules on a hydrophobic plate. Phospholipids tested were not recognized by TNM-FL. Data represent means of three independent experiments. Error bars, s.d. (B–D) HeLa cells were treated with fluorescein-5-thiosemicarbazide (B) or TNM-FL (C, D) on ice for 30 min. After excess probe molecules were washed out, cells were observed under microscopy. Magnified images of C are shown in D. Scale bars, 50 µm. (TIF) [file pone.0083716.s005.tif]
